# Complete Chloroplast Genome of *Cardamine microzyga* and Phylogenetic Analysis

**DOI:** 10.1002/ece3.73951

**Published:** 2026-07-19

**Authors:** Yongming Fan, Yan Wu, Heng Zeng, Can Zhao, Chu Li, Le Chen, Jian Hu, Lingjun Li

**Affiliations:** ^1^ School of Human Settlements North China University of Water Resources and Electric Power Zhengzhou China; ^2^ Hydrology and Water Resources Monitoring Center for the Upper Reaches of Ganjiang River Ganzhou China; ^3^ College of Landscape Architecture Beijing Forestry University Beijing China; ^4^ Bureau of Comprehensive Development Ministry of Water Resources Beijing China

**Keywords:** *Cardamine microzyga*, chloroplast genome, nucleotide diversity, phylogenetic analysis, simple sequence repeats

## Abstract

*Cardamine microzyga* (Brassicaceae) is a perennial herbaceous species native to mountainous regions of southwestern China, yet its genomic resources remain poorly characterized. In this study, we assembled and annotated the complete chloroplast (cp) genome of *C. microzyga* based on Illumina HiSeq 4000 sequencing data, and conducted comprehensive analyses of its genomic structure, repeat sequences, codon usage, nucleotide diversity, and phylogenetic relationships. The cp genome is 154,661 bp in length, exhibiting a typical quadripartite structure comprising a large single‐copy (LSC) region (84,261 bp), a small single‐copy (SSC) region (17,774 bp), and a pair of inverted repeat (IR) regions (26,313 bp each). A total of 129 genes were annotated, including 84 protein‐coding genes, 37 tRNA genes, and 8 rRNA genes. Fifty‐seven mononucleotide and four dinucleotide simple sequence repeats (SSRs) were identified, all of which exhibited a pronounced A/T bias, together with nine complex compound SSRs. Comparative analysis of the IR/LSC and IR/SSC boundaries among 11 Brassicaceae species revealed high structural conservation, with only minor variations. Codon usage analysis indicated that 30 codons had relative synonymous codon usage (RSCU) values > 1, with a strong preference for A/T‐ending codons. Nucleotide diversity (Pi) analysis across 106 protein‐coding genes identified nine hypervariable regions (Pi > 0.0156), among which *trnS‐GCU* (Pi = 0.03736), *rrn4.5S* (0.03280), and *ycf1* (0.03224) represented promising candidates for DNA barcoding and phylogenetic marker development. Phylogenetic reconstruction based on 45 complete cp genomes using maximum likelihood (ML) method placed *C. microzyga* within a well‐supported clade (bootstrap = 100) comprising *C. lyrata, C* *. fallax* *, and C. amariformis,* with *Rorippa* as the sister group. This study provides the first complete cp genome of *C. microzyga*, which not only fills a key genomic gap in an alpine‐endemic species but also offers insights into the adaptive evolution of high‐altitude plants. These resources will facilitate future taxonomic, evolutionary, and conservation genetics studies in the genus *Cardamine* and the family Brassicaceae.

## Graphical abstract

This study reports the first complete chloroplast genome of Cardamine microzyga, a perennial herb from mountainous southwestern China. The genome is 154,661 bp in length with a typical quadripartite structure and contains 129 genes. Comparative genomic analyses identified several hypervariable regions (e.g., trnS‐GCU, rrn4.5S, ycf1) as promising DNA barcodes and phylogenetic markers. Maximum likelihood phylogeny based on 45 chloroplast genomes placed C. microzyga in a well‐supported clade with *C. lyrata* , *C. fallax* , and C. amariformis, with Rorippa as the sister group.

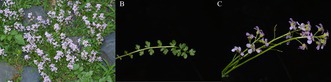

## 1 Introduction

The Brassicaceae family is one of the largest families of angiosperms, comprising approximately 321 genera and 3660 species, widely distributed in temperate and cold regions worldwide (Al‐Shehbaz 2012). This family not only includes a variety of economically important crops but also attracts considerable attention due to its unique status in phylogenetic, molecular evolutionary, and comparative genomic studies. *Cardamine* is one of the largest genera in the Brassicaceae, with about 160–200 species globally, widely distributed in the Northern temperate zone and mountainous regions (Carlsen et al. 2009). The species of this genus exhibit diverse morphology and rich ecological adaptability, making them an ideal group for studying species differentiation, polyploidization, and adaptive evolution (Zozomová‐Lihová et al. 2025). *Cardamine microzyga* is a perennial herbaceous plant mainly distributed in alpine regions of Yunnan and Sichuan, possessing important ecological and conservation value (Schulz 1903). However, genomic resources for this species are extremely scarce, and its phylogenetic position and evolutionary history remain unclear, which limits in‐depth exploration of its genetic diversity and adaptive mechanisms.

Due to its maternal inheritance, conserved structure, and moderate evolutionary rate, the chloroplast genome has been widely used in plant phylogenetic reconstruction, species identification, and population genetics studies (Cauz‐Santos 2025). With the development of high‐throughput sequencing technologies, the cost of chloroplast genome sequencing has significantly decreased, and an increasing number of chloroplast genomes have been sequenced, providing important data support for plant comparative genomics and evolutionary biology research (Li et al. 2020). To date, chloroplast genomes of several species in the genus *Cardamine* have been reported (Raman and Park 2021; Xu et al. 2022), but the chloroplast genome of *C. microzyga* has not yet been documented. Its genomic structural characteristics, repeat sequence distribution, codon usage bias, and comparative genomic information with closely related species remain unknown.

**FIGURE 1:**
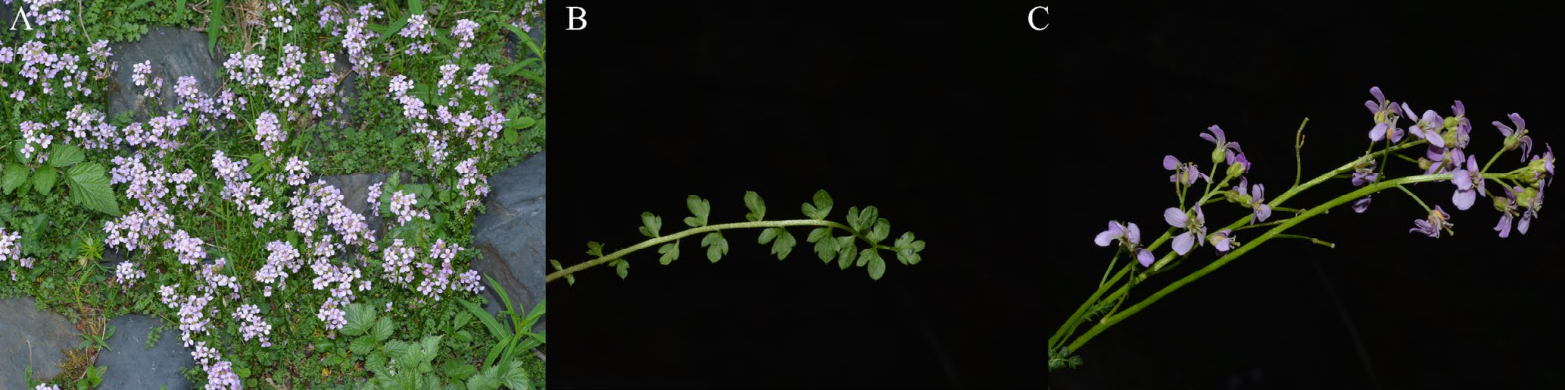
*C. microzyga*. (A) Plant habit; (B) cauline leaves; (C) flower. Photo credit: Associate Research Professor Juntong Chen, Kunming Institute of Botany, Chinese Academy of Sciences.

*C. microzyga* is a perennial herb with a creeping rhizome bearing numerous slender fibrous roots. The cauline leaves are 2–3 in number, with 6–10 pairs of leaflets, usually without petiolules. The inflorescence is a terminal raceme with many flowers (Figure 1). In this study, we sequenced, assembled, and annotated the complete chloroplast genome of *C. microzyga* for the first time using the Illumina HiSeq 4000 high‐throughput sequencing platform. This work addresses a critical gap in genomic resources for alpine Brassicaceae and provides a foundation for understanding high‐altitude adaptation and genetic diversity patterns. Based on this, we systematically analyzed the genome structure, simple sequence repeats (SSRs), codon usage bias, nucleotide diversity, and IR/SC boundary variation. Furthermore, combined with chloroplast genome data from 44 Brassicaceae species, we constructed a high‐resolution phylogenetic tree with the following aims: (1) to reveal the structural characteristics and evolutionary patterns of the *C. microzyga* chloroplast genome; (2) to identify hypervariable regions and SSR loci as candidate resources for future molecular marker development; and (3) to clarify the phylogenetic position of *C. microzyga* within the genus *Cardamine* and the family Brassicaceae. The results of this study will provide an important genomic foundation for taxonomic, evolutionary biological, and genetic resource conservation research on the genus *Cardamine*.

## 2 Materials and Methods

### 2.1 Plant Material and DNA Extraction and Sequencing

Leaf samples of *C. microzyga* were collected from Gongshan County, Yunnan Province, China. No specific permits were required for this collection. The species was authenticated by Associate Researcher Juntong Chen at the Kunming Institute of Botany, Chinese Academy of Sciences. The collected leaf material was stored at −80°C in the Aquatic Plant Application and Germplasm Enhancement Laboratory of the Yellow River Basin, North China University of Water Resources and Electric Power, for subsequent DNA isolation. A voucher specimen was deposited in the same facility under the accession number NCWU‐20200617. Using the magnetic bead‐based approach, total genomic DNA was extracted from fresh leaf tissue with the CretMag Multi Sample DNA Kit (Suzhou Cretaceous Biotechnology Co. Ltd., Suzhou, China), strictly adhering to the manufacturer's instructions (Fan et al. 2025). The quality of the extracted DNA was evaluated, yielding a concentration of 34.5 ng/μL as measured by a Qubit fluorometer and an A260/280 ratio of 1.78 determined via NanoDrop spectrophotometry. Additionally, agarose gel electrophoresis revealed an intact DNA band with no visible degradation, confirming its suitability for subsequent applications. Library preparation was carried out with the VAHTS Universal Plus DNA Library Prep Kit, and paired‐end sequencing (150 bp) was conducted on an Illumina HiSeq 4000 platform (Shanghai OE Biotech Co. Ltd., Shanghai, China). Raw sequencing data underwent quality control using FastQC software (version 0.12.0; available at: http://www.bioinformatics.babraham.ac.uk/projects/fastqc/; accessed on December 1, 2024).

### 2.2 Complete Genome Assembly and Annotation

Raw sequencing reads were first filtered using fastp v0.23.4 (https://github.com/OpenGene/fastp) to eliminate adapter sequences and low‐quality bases, yielding clean reads for downstream analysis (Chen et al. 2018; Chen 2025). The complete chloroplast genome of *C. microzyga* was then assembled with GetOrganelle v1.7.7.1 (https://github.com/Kinggerm/GetOrganelle) under default parameters (Jin et al. 2020), employing the chloroplast genome of *C. amariformis* (NCBI accession MZ043776.1) as the reference. Genome annotation, including the identification of protein‐coding genes, transfer RNAs, and ribosomal RNAs, was performed using the CPGAVAS2 web server (http://www.herbalgenomics.org/cpgavas/) (Shi et al. 2019). A circular map of the chloroplast genome was generated with OGDRAW v1.2 (http://ogdraw.mpimp‐golm.mpg.de/) (Lohse et al. 2007). All online tools were accessed on 18 December 2025.

### 2.3 Identification of Simple Sequence Repeats (SSRs)

SSRs in the *C. microzyga* chloroplast genome were detected using the MISA web server (https://webblast.ipk‐gatersleben.de/misa/) (Beier et al. 2017). The minimum threshold numbers of repeat units were configured as 10 for mono‐nucleotide, 6 for di‐nucleotide, and 5 for tri‐, tetra‐, penta‐, and hexa‐nucleotide SSR motifs, following the parameters established in previous studies (Fan et al. 2025). The online tool was accessed on 19 December 2025 for this analysis.

### 2.4 Comparative Genomic Analysis

To investigate structural variation and sequence divergence, the chloroplast genome of *C. microzyga* was compared with those of nine additional *Cardamine* species and two Rorippa species: *C. heptaphylla* (NC_060864.1), *C. fallax* (MZ043778.1), *C. bulbifera* (MT136868.1), *C. amariformis* (MZ043776.1), *C. pentaphyllos* (MN651500.1), *C. macrophylla* (MF405340.1), *C. libagouensis* (PX328981.1), *Rorippa palustris* (OR124737.1), and *R. sylvestris* (PP297068.1). The chloroplast genome of *Lepidium didymum* (PV712662.1) was used as the reference for sequence alignment. Whole‐genome alignments were performed with the mVISTA program in Shuffle‐LAGAN mode (Frazer et al. 2004). Genome sequences of the nine non‐reference species were retrieved from the NCBI database; together with the newly assembled *C. microzyga* genome, they were aligned against the *L. didymum* reference. Nucleotide diversity (Pi) across the aligned chloroplast genomes was calculated using CPStools v2.5 (Huang et al. 2024), providing insight into the most variable regions among these taxa.

### 2.5 Codon Usage Analysis

The pattern of codon usage bias in the *C. microzyga* chloroplast genome was examined using CodonW v1.4.2 (https://sourceforge.net/projects/codonw/). Relative synonymous codon usage (RSCU) values were calculated for each protein‐coding sequence to evaluate synonymous codon preferences (Sharp and Li 1987). By conventional interpretation, an RSCU value of 1.00 indicates no bias toward a particular codon, while values exceeding 1.00 denote preferential or more frequent usage of that codon. The analysis was performed in 19 December 2025.

### 2.6 Phylogenetic Analysis

To determine the phylogenetic position of *C. microzyga* within the Brassicaceae, a maximum likelihood analysis was conducted based on complete chloroplast genome sequences from 45 taxa. The dataset comprised the newly assembled *C. microzyga* genome and 44 additional sequences retrieved from the NCBI database, representing the genera *Aethionema*, *Brassica*, *Chorispora*, *Malcolmia*, *Hesperis*, *Lepidium*, *Barbarea*, *Rorippa*, and *Cardamine*. *Aethionema arabicum* (NC_034367.1) was designated as the outgroup. All analyses were performed using the PhyloSuite v1.2.3 platform, which integrates multiple tools for phylogenomic data processing (Xiang et al. 2023). Whole chloroplast genome sequences were aligned with MAFFT v7.505 under the auto strategy, which automatically selects the optimal alignment algorithm based on sequence characteristics (Katoh et al. 2019). The resulting alignments were refined using trimAl v1.2 to eliminate poorly aligned regions and gaps (Capella‐Gutiérrez et al. 2009). For protein‐coding genes, batch optimization was performed with MACSE v2.0.3 to ensure accurate codon‐aware alignment. Phylogenetic inference was implemented in IQ‐TREE (http://www.iqtree.org/) using the maximum likelihood method, and node support was assessed through 5000 bootstrap replicates (Nguyen et al. 2015).

## 3 Results

### 3.1 Cp Genome Features of *C. microzyga*

**FIGURE 2:**
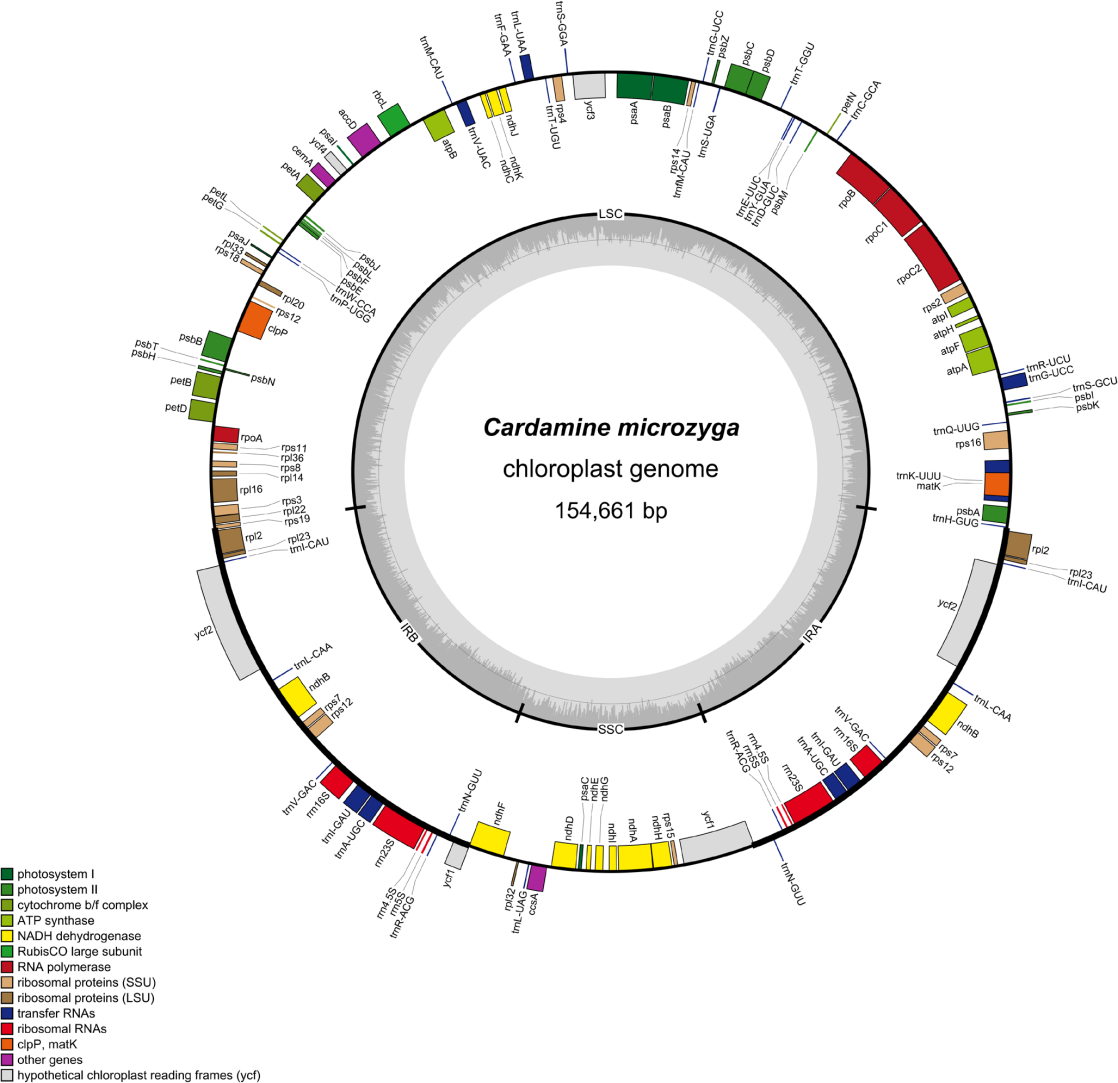
The circular complete cp genome map of *C. microzyga*. Genes shown outside the outer circle are transcribed clockwise, whereas those shown inside are transcribed counterclockwise.

Sequencing of the *C. microzyga* chloroplast genome generated 51,342,214 raw reads, corresponding to 7.70 Gb of raw data. Following quality filtering, 51,265,436 clean reads were retained, amounting to 7.63 Gb of clean data, with 96.06% of bases achieving a quality score above Q30 (representing a base‐calling error probability below 0.1%). The complete chloroplast genome of *C. microzyga* is a circular molecule of 154,661 bp in length, exhibiting the typical quadripartite structure characteristic of most angiosperm chloroplast genomes (Figure 2). This architecture comprises a large single‐copy (LSC) region of 84,261 bp, a small single‐copy (SSC) region of 17,774 bp, and a pair of inverted repeat regions (IRa and IRb), each spanning 26,313 bp. The overall GC content of the genome is 37.87%. Consistent with patterns observed in other angiosperms, the IR regions display elevated GC content (42.37% for both IRa and IRb) compared to the single‐copy regions, with the LSC and SSC regions exhibiting GC contents of 34.00% and 29.26%, respectively.

Genome annotation revealed a total of 129 genes in the *C. microzyga* chloroplast genome, comprising 84 protein‐coding genes, 37 transfer RNA (tRNA) genes, and 8 ribosomal RNA (rRNA) genes (Table 1). Based on their predicted functions, these genes were categorized into three main groups: photosynthesis‐related genes, self‐replication‐related genes, and other genes (Table 1). The photosynthesis category contained 44 genes, including subunits of photosystem I (5 genes), photosystem II (15 genes), NADH dehydrogenase (12 genes), cytochrome b/f complex (6 genes), ATP synthase (5 genes), and the large subunit of Rubisco (rbcL). The self replication category comprised 74 genes, encompassing proteins of the large ribosomal subunit (11 genes) and proteins of the small ribosomal subunit (14 genes), subunits of RNA polymerase (4 genes), ribosomal RNAs (8 genes), and transfer RNAs (37 genes). The remaining 11 genes were classified as other genes, including *matK* (maturase), *clpP* (protease), *cemA* (envelope membrane protein), *accD* (acetyl‐CoA carboxylase), *ccsA* (c‐type cytochrome synthesis gene), and six conserved open reading frames (*ycf1*, *ycf2*, *ycf3*, *ycf4*).

**TABLE 1:** Gene composition in *C. microzyga* chloroplast genome.

Category	Gene group	Gene name	Number
Photosynthesis	Subunits of photosystem I	*psaA, psaB, psaC, psaI, psaJ*	5
	Subunits of photosystem II	*psbA, psbK, psbI, psbM, psbD, psbC, psbZ, psbJ, psbL, psbF, psbE, psbB, psbT, psbN, psbH*	15
	Subunits of NADH‐dehydrogenase	*ndhJ, ndhK, ndhC, ndhB(2)#, ndhF, ndhD, ndhE, ndhG, ndhI, ndhA#, ndhH*	12
	Subunits of cytochrome b/f complex	*petN, petA, petL, petG, petB#, petD#*	6
	Subunits of ATP synthase	*atpA, atpF#, atpH, atpI, atpB*	5
	Large subunit of rubisco	*rbcL*	1
Self replication	Proteins of large ribosomal subunit	*rpl33, rpl20, rpl36, rpl14, rpl16#, rpl22, rpl2(2)#, rpl23(2), rpl32*	11
	Proteins of small ribosomal subunit	*rps12(2)##, rps16#, rps2, rps14, rps4, rps18, rps11, rps8, rps3, rps19, rps7(2), rps15*	14
	Subunits of RNA polymerase	*rpoC2, rpoC1#, rpoB, rpoA*	4
	Ribosomal RNAs	*rrn16S(2), rrn23S(2), rrn4.5S(2), rrn5S(2)*	8
	Transfer RNAs	*trnH‐GUG, trnK‐UUU#, trnQ‐UUG, trnS‐GCU, trnG‐UCC(2)#, trnR‐UCU, trnC‐GCA, trnD‐GUC, trnY‐GUA, trnE‐UUC, trnT‐GGU, trnS‐UGA, trnfM‐CAU, trnS‐GGA, trnT‐UGU, trnL‐UAA#, trnF‐GAA, trnV‐UAC#, trnM‐CAU, trnW‐CCA, trnP‐UGG, trnI‐CAU(2), trnL‐CAA(2), trnV‐GAC(2), trnI‐GAU(2)#, trnA‐UGC(2)#, trnR‐ACG(2), trnN‐GUU(2), trnL‐UAG*	37
Other genes	Maturase	*matK*	1
	Protease	*clpP##*	1
	Envelop membrane protein	*cemA*	1
	Acetyl‐CoA carboxylase	*accD*	1
	c‐type cytochrom synthesis gene	*ccsA*	1
	Conserved open reading frames	*ycf3##, ycf4, ycf2(2), ycf1(2)*	6

*Note:* #Intron number, (n): Gene copy number.

Intron analysis identified 17 distinct genes harboring introns within the *C. microzyga* chloroplast genome (Table S1). Among these, two genes exhibited complex structures with multiple introns: *ycf3* and *clpP* each contained two introns. The remaining 15 genes possessed a single intron each: *trnK‐UUU*, *rps16*, *trnG‐UCC*, *atpF*, *rpoC1*, *trnL‐UAA*, *trnV‐UAC*, *petB*, *petD*, *rpl16*, *rpl2*, *ndhB*, *trnI‐GAU*, *trnA‐UGC*, and *ndhA*. Notably, four of these single‐intron genes (*rpl2*, *ndhB*, *trnI‐GAU*, *trnA‐UGC*) were located within the IR regions and therefore existed as two copies, each present on both the positive and negative strands (Table S1). The complete chloroplast genome of *C. microzyga* that support the findings of this study have been deposited into China National GeneBank Sequence Archive (CNSA) under accession number CNX1361155.

### 3.2 Analysis of SSRs

**FIGURE 3:**
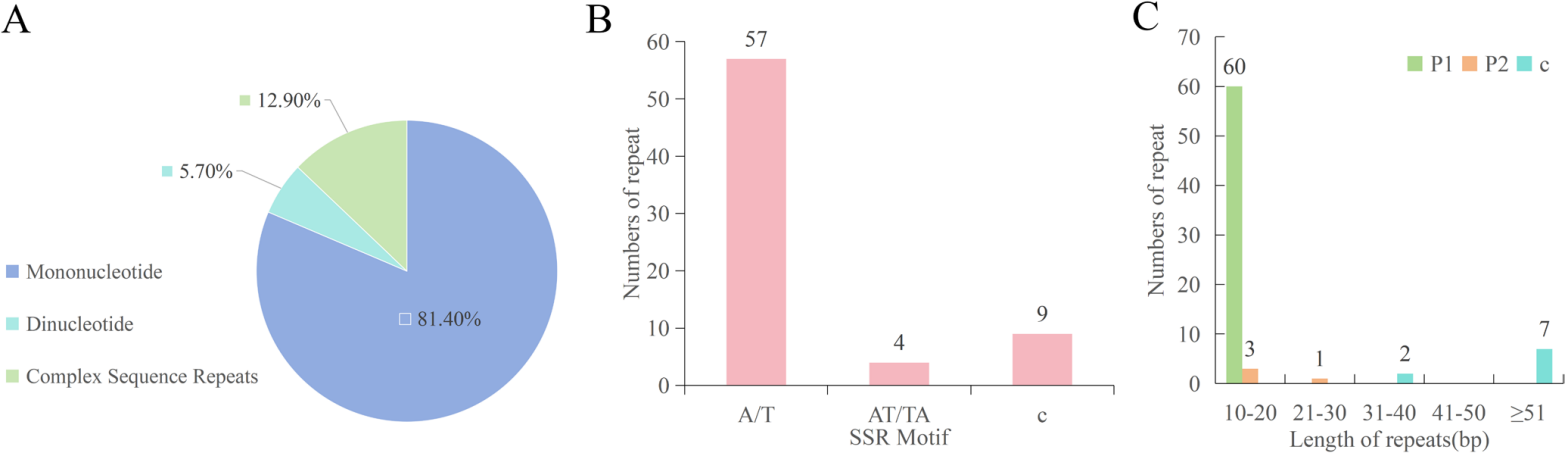
Analysis of repeat sequences in the *C. microzyga* chloroplast genome. (A) Composition and proportion of SSR types, (B) Distribution of different SSR repeat motifs, (C) Statistical count of SSR numbers by type.

In the chloroplast genome of *C. microzyga*, a total of 70 SSR loci were identified, with lengths ranging from 10 to 146 bp (Figure 3 and Table S2). Mononucleotide repeats were the predominant type, accounting for 57 (81.4%) of the total SSRs. All mononucleotide repeats were composed exclusively of A or T bases, reflecting a strong A/T bias within the chloroplast genome. Dinucleotide repeats comprised four (5.7%) loci, all of which were of the AT/TA type. In addition, nine complex compound SSRs (12.9%) were detected, exhibiting diverse structural configurations and considerable length variation. Among these, several compound SSRs exceeded 100 bp in length, with the largest spanning 146 bp (ID 4). Notably, one compound SSR (ID 59) featured a multi‐motif composition of (A)10(AT)6*aa(T)11, representing a distinctive repeat architecture that incorporates both mononucleotide and dinucleotide units separated by short spacer sequences (Table S2).

Length distribution analysis revealed that the majority of SSRs (61 of 70) were concentrated within the 10–22 bp range, consistent with typical short‐fragment SSR characteristics observed in angiosperm chloroplast genomes. Within this range, mononucleotide repeats were the most abundant, followed by a small number of dinucleotide repeats. Larger SSRs exceeding 50 bp were exclusively associated with compound repeat structures, which contributed substantially to the overall length diversity and mutational potential of SSR loci in this genome.

### 3.3 Comparative Analysis of Genome Structure in *C. microzyga* and Its Related Species

**FIGURE 4:**
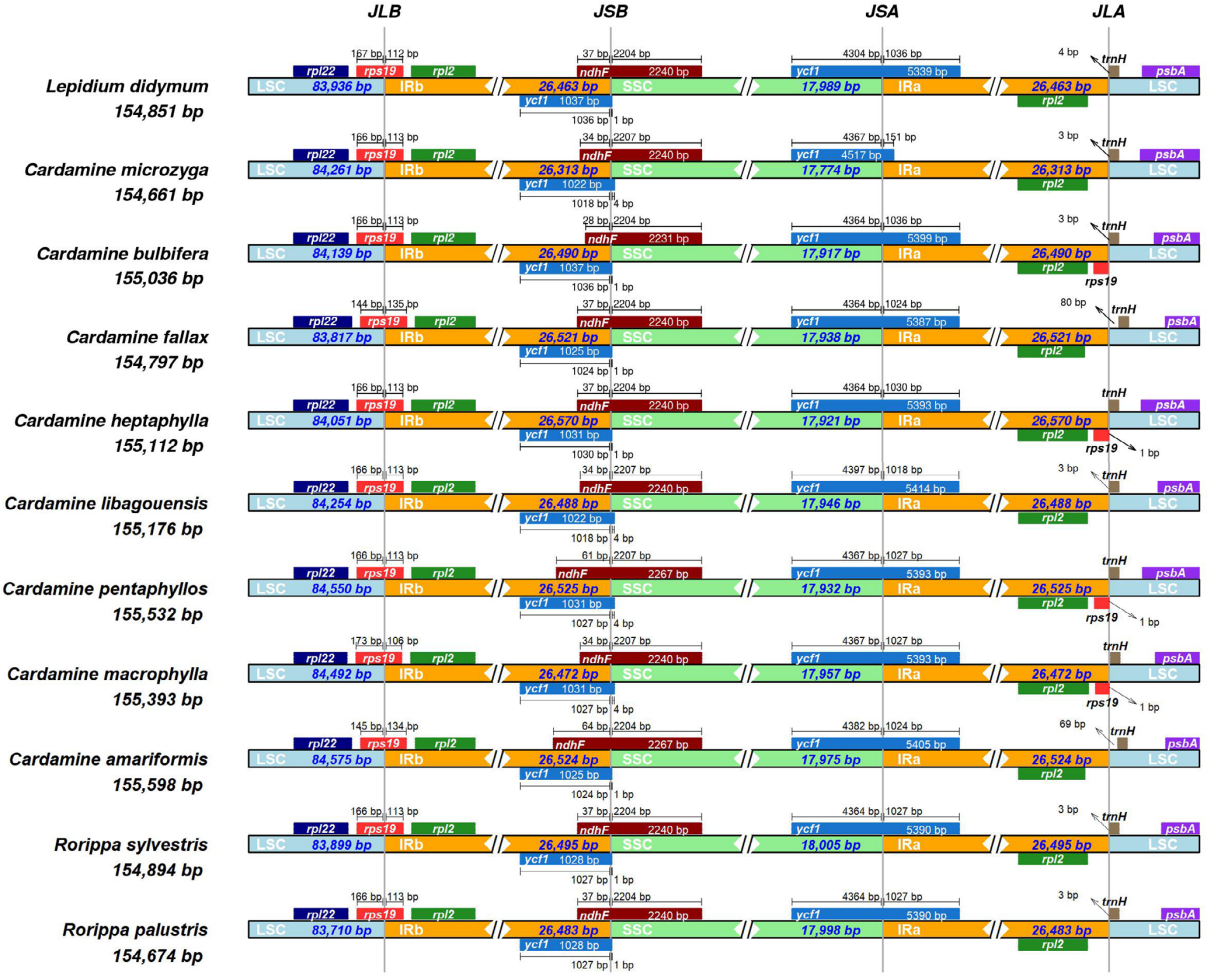
Comparison of the LSC, SSC, and IR boundary regions among 11 chloroplast genomes of Brassicaceae species. The gaps between the genes and the boundaries are indicated by the base lengths. Genes are denoted by colored boxes. JLA, IRa/LSC junction; JLB, LSC/IRb junction; JSA, SSC/IRa junction; JSB, IRb/SSC junction.

Comparative analysis of the IR/SSC and IR/LSC boundaries can precisely locate the expansion and contraction events of the IR regions, thereby revealing the dynamic evolutionary history of chloroplast genomes. To further elucidate the structural evolutionary history of the chloroplast genome of *C. microzyga*, we performed comparative analyses of the IR/SSC and IR/LSC boundaries among 10 representative Brassicaceae species along with the target species *C. microzyga*, including *L. didymum* , *C. bulbifera* , *C. fallax* , *C. heptaphylla*, *C. libagouensis*, *C. pentaphyllos*, *C. macrophylla* , *C. amariformis*, *Rorippa sylvestris* , and *R. palustris* (Figure 4). The genes involved in the IR/SSC and IR/LSC boundary regions include *ycf1*, *ndhF*, *rps19*, *rpl2*, *trnH*, *psbA*, and *rpl22*. In *C. microzyga* and its closely related species, the length of the IR region ranges from 26,313 to 26,570 bp, exhibiting high conservation with only slight length variation.

In *C. microzyga* and all other analyzed Brassicaceae species, the *ycf1* gene spanned the IRb/SSC boundary and was predominantly located within the IRb region. Simultaneously, the *ycf1* gene also spanned the SSC/IRa boundary and was predominantly located within the SSC region. In *L. didymum* , the *ycf1* gene followed an identical boundary‐spanning pattern, with 1037 bp in the IRb and 4304 bp in the SSC, suggesting a conserved structural arrangement of this gene across Brassicaceae (Figure 4).

The length of the ndhF gene ranges from 2240 to 2267 bp. In all 11 species, this gene is located at the SSC/IRa boundary, with the majority situated in the SSC region and a small portion extending into the IRb region. With the exception of *C. bulbifera* , *C. pentaphyllos*, *C. macrophylla* , and *C. heptaphylla*, a functional copy of the *rps19* gene was present at the LSC/IRb junction in *C. microzyga* and all other close relatives, these four exceptional species harbored an additional copy of *rps19* at the IRa/LSC junction. The *rpl2* gene was completely located within the IR region and distant from both the LSC/IRb and IRa/LSC junctions in all 11 species (Figure 4).

The *trnH* gene was generally located within the LSC region at the IRa/LSC junction, with distances to the boundary ranging from 3 to 80 bp across most species. In *C. microzyga*, *trnH* was positioned 3 bp away from the IRa/LSC boundary, consistent with the majority of *Cardamine* and *Rorippa* species. The *psbA* gene was universally located within the LSC region, adjacent to *trnH*. The *rpl22* gene was consistently located within the LSC region at the LSC/IRb junction (Figure 4).

**FIGURE 5:**
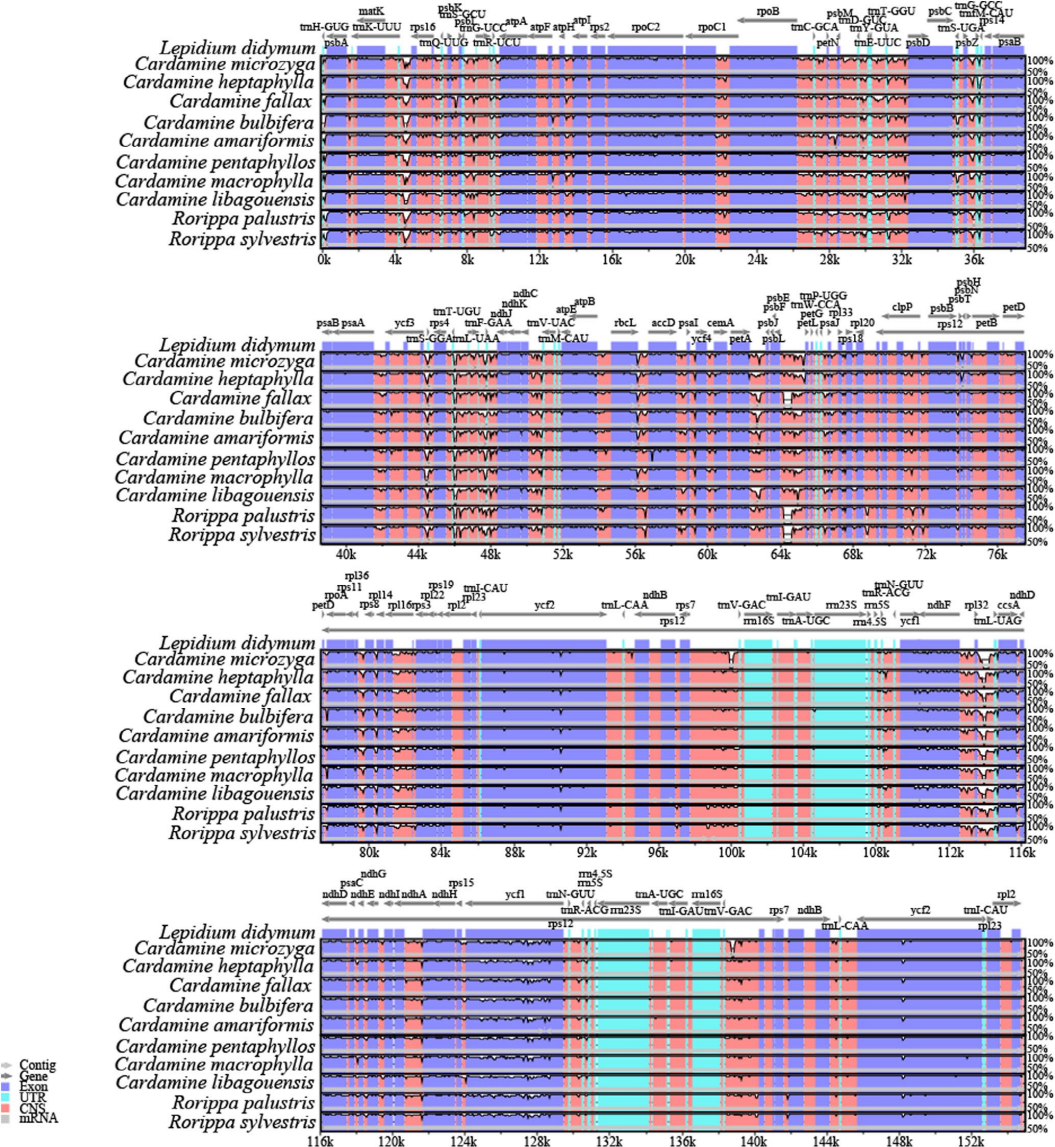
Sequence alignment of 11 Brassicaceae cp. genomes using the mVISTA program with *L. didymum* as a reference. X‐axis: The coordinates in the cp. genome, Y‐axis: Percent identity within 50%–100%. The transcriptional direction of genes indicated by gray arrows. Regions where the identity curve falls to the baseline (~50%) indicate sequences that are highly divergent or absent in the query genome. The gene names are indicated directly above the dark gray arrows.

To clarify the sequence conservation characteristics of the chloroplast genomes of *C. microzyga* and its related genera within Brassicaceae, this study performed a global alignment of the chloroplast genomes of 11 species using the mVISTA tool (Figure 5). With *L. didymum* as the reference sequence, the results indicate that the overall structure and gene arrangement of the chloroplast genomes of these species are highly consistent. However, the coding regions exhibited significantly higher sequence conservation compared to the non‐coding regions.

### 3.4 Analysis of Codon Usage Bias in the Cp Genome of *C. microzyga*

The relative synonymous codon usage (RSCU) values were calculated based on the protein‐coding sequences of the *C. microzyga* chloroplast genome (Table S3). The chloroplast genome contains a total of 64 codons, of which 61 codons encode 20 amino acids and three are stop codons. In the *C. microzyga* chloroplast genome, leucine (Leu), arginine (Arg), and serine (Ser) are the most abundant amino acids, while methionine (Met) and tryptophan (Trp) are the least abundant.

A total of 30 codons had RSCU values greater than 1, indicating that their usage frequency is higher than the theoretical average under the assumption of no codon usage bias; 32 codons had RSCU values less than 1, indicating that their usage frequency is lower than the theoretical average. Methionine (Met) and tryptophan (Trp) are each encoded by only one codon, and their RSCU values equal 1, reflecting the absence of synonymous codon usage bias for these two amino acids. Among the three stop codons, TAA (RSCU = 2.00) is the most frequently used, followed by TAG (0.76) and TGA (0.24), indicating that TAA is the preferred translation termination signal.

### 3.5 Nucleotide Diversity Analysis

**FIGURE 6:**
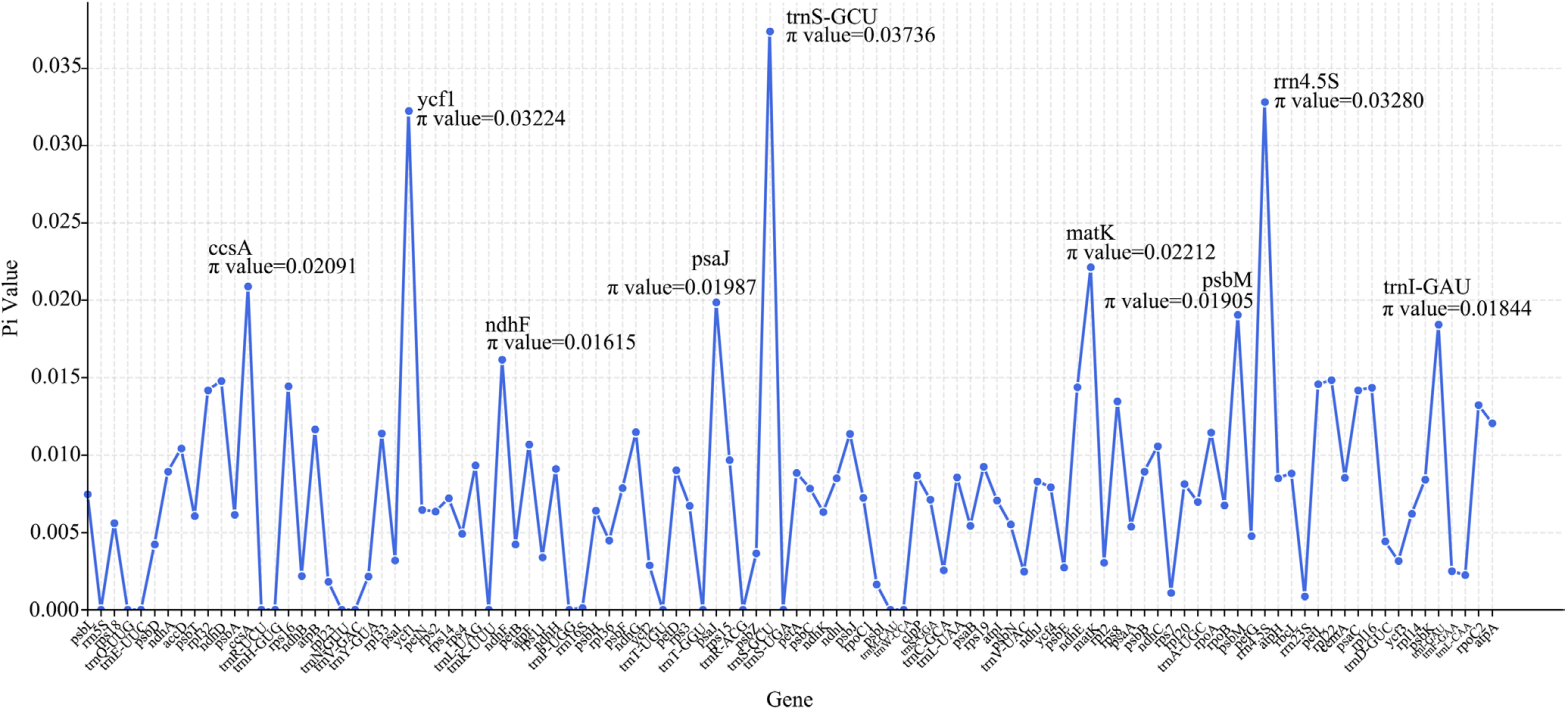
Nucleotide diversity (π) values of chloroplast coding regions in *C. microzyga* and its 10 related species.

To investigate the nucleotide variation in the chloroplast genome of *C. microzyga* and its closely related species, we performed a sliding window analysis to identify regions with high sequence divergence. Nucleotide diversity (Pi) values were calculated for 106 protein‐coding genes from 11 species to comprehensively understand sequence conservation and variation patterns (Figure 6). Among the analyzed coding regions, Pi values ranged from 0.00,000 to 0.03,736, with an average of 0.0,078. The threshold of 0.0,156 was set as twice the average Pi value (0.0,078) to identify genes with substantially higher than average nucleotide diversity. A total of nine hypervariable genes with Pi values exceeding 0.0,156 were identified: *trnS‐GCU* (0.03,736), *rrn4.5S* (0.03,280), *ycf1* (0.03,224), *matK* (0.02,212), *ccsA* (0.02,091), *psaJ* (0.01,987), *psbM* (0.01,905), *trnI‐GAU* (0.01,844), and *ndhF* (0.01,615). Among these, *trnS‐GCU* exhibited the highest nucleotide diversity and represents a potential hotspot for molecular marker development. Notably, 15 genes were completely conserved (Pi = 0.00,000) across the compared species, including *rrn5S* as well as *trnQ‐UUG*, *trnE‐UUC*, *trnR‐UCU*, *trnH‐GUG*, *trnN‐GUU*, *trnV‐GAC*, *trnK‐UUU*, *trnP‐UGG*, *trnT‐UGU*, *trnT‐GGU*, *trnR‐ACG*, *trnS‐UGA*, *trnW‐CCA*, and *trnM‐CAU*, reflecting the high conservation of these genes in the *Cardamine* chloroplast genome. The identification of these hypervariable regions provides valuable candidate markers for future phylogenetic studies and DNA barcoding applications in the genus *Cardamine* and related Brassicaceae taxa.

### 3.6 Phylogenetic Tree Construction and Analysis of Species Clusters

**FIGURE 7:**
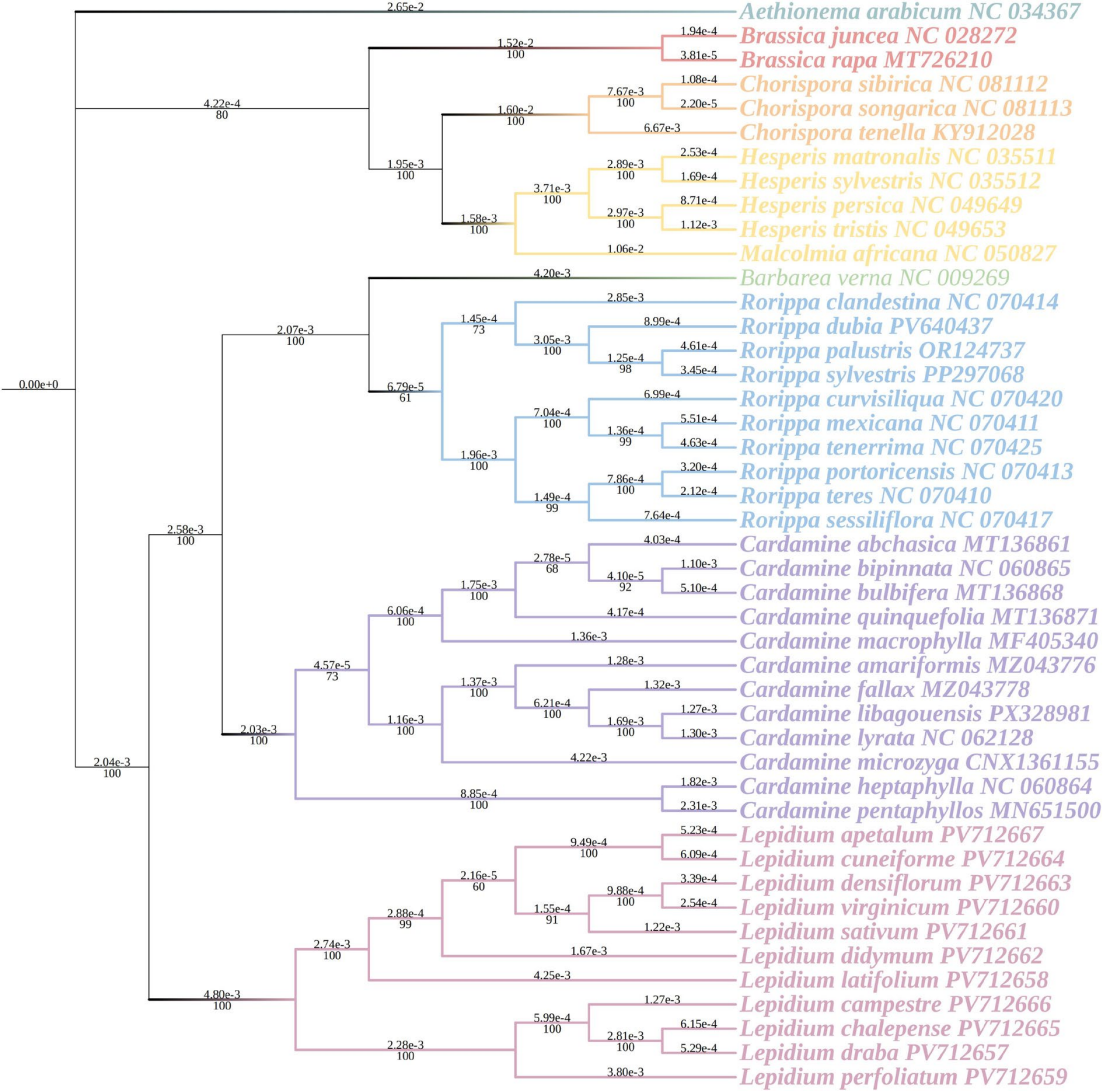
ML phylogenetic tree of 45 complete cp genomes. Bootstrap values and branch lengths are shown near each node. Different colors represent the differences in the clustering results. Colors in the phylogenetic tree represent different clades.

To clarify the phylogenetic position and evolutionary relationships of *C. microzyga* within the genus *Cardamine* and the family Brassicaceae, this study constructed a maximum likelihood (ML) phylogenetic tree based on complete chloroplast genome sequences of 45 representative Brassicaceae species, with *Aethionema arabicum* as the outgroup (Figure 7). The topology of the ML tree strongly supported the monophyly of the genus *Cardamine*, which formed a highly supported clade (bootstrap value = 100), indicating close evolutionary relationships among species within the genus.

Within the *Cardamine* clade, three major subclades (Clade A, Clade B, and Clade C) were identified, which is consistent with previous studies (Hu et al. 2015; Raman and Park 2021, 2022; Raman et al. 2021; Xu et al. 2022). Clade A includes species such as *C. abchasica* , *C. quinquefolia* , *C. bipinnata* , *C. bulbifera* , and *C. macrophylla* . Clade B comprises *C. lyrata* , *C. fallax* , *C. amariformis*, *C. libagouensis*, and the target species *C. microzyga*, which together form a highly supported subclade (bootstrap value = 100). Clade C includes *C. pentaphyllos* and *C. heptaphylla*.

It is worth noting that *C. microzyga* is closely related to *C. lyrata* , *C. fallax* , and *C. amariformis* within Clade B, indicating that they share a relatively recent common ancestor. This finding is consistent with previous phylogenetic studies on the genus *Cardamine*. Furthermore, the *Cardamine* clade forms a sister group to the *Rorippa* clade, and together with the *Barbarea* clade they constitute a larger branch, revealing a close evolutionary relationship among these three genera. Most branches in the ML tree have high bootstrap support values (60–100), confirming the reliability of the phylogenetic inference in this study. These results not only clarify the systematic position of *C. microzyga* within the genus *Cardamine*, but also provide valuable references for studies on the evolutionary history of the Brassicaceae, laying a foundation for future taxonomic and evolutionary research.

## 4 Discussion

This study reports, for the first time, the complete chloroplast genome sequence of *C. microzyga*, and systematically analyzes its structural features, repetitive sequences, codon usage bias, nucleotide diversity, and phylogenetic relationships. The findings not only enrich the chloroplast genome resources of the genus *Cardamine* but also provide important foundations for understanding the evolutionary history of species within this genus and for developing molecular markers.

The complete chloroplast genome of *C. microzyga* is 154,661 bp in length and exhibits a typical quadripartite structure, consisting of a pair of IR regions (each 26,313 bp), one LSC region (84,261 bp), and one SSC region (17,774 bp) (Figure 2). This genome size is highly consistent with those reported for other *Cardamine* species ( *C. fallax* 154,797 bp, *C. occulta* 154,796 bp) (Raman and Park 2021; Raman et al. 2021), indicating that the chloroplast genome size within the genus *Cardamine* is highly conserved. The GC content of the IR regions (42.37%) is significantly higher than that of the LSC region (34.00%) and the SSC region (29.26%), which is consistent with the characteristics of most angiosperm chloroplast genomes (Zhang et al. 2024; Liu et al. 2025). A total of 129 genes were annotated, and the presence of conserved open reading frames such as *ycf1* and *ycf2* further confirms the high conservation of gene composition in the chloroplast genome (Kadam et al. 2025). Intron analysis revealed that a total of 17 genes in the *C. microzyga* chloroplast genome contain introns, among which *ycf3* and *clpP* each contain two introns, a feature commonly observed in the *Brassicaceae* family (Ren et al. 2018; Xu et al. 2022). The four single‐intron genes located in the IR regions (*rpl2*, *ndhB*, *trnI‐GAU*, and *trnA‐UGC*) are each present in two copies, which is consistent with the repetitive nature of the IR regions (Table S1). The presence of introns may regulate gene expression through alternative splicing and play important roles in plant adaptation to different environments (Zhu et al. 2025).

Comparative analysis of the IR/SSC boundaries is an effective approach to reveal the structural evolution of chloroplast genomes (Li et al. 2025). In this study, we compared the boundary regions of 11 Brassicaceae species, and the results showed that the lengths of the IR regions ranged from 26,313 to 26,570 bp, indicating a high overall conservation (Figure 4). The *ycf1* gene spanned both the IRb/SSC and SSC/IRa boundaries in all examined species, and its distribution proportions in the IRb and SSC regions were relatively stable, which is consistent with previous findings that *ycf1* is one of the genes most prone to boundary expansion/contraction in chloroplast genomes (Wang et al. 2025; Guo et al. 2025). Furthermore, an additional copy of the *rps19* gene was observed in *C. bulbifera* , *C. pentaphyllos*, *C. macrophylla* , and *C. heptaphylla*, suggesting that these species may have experienced specific IR expansion events. Similar phenomena have also been reported in the Fabaceae and Poaceae families (Hao et al. 2025). In *C. microzyga*, the *trnH* gene is located only 3 bp away from the IRa/LSC boundary. This feature is consistent with that of most *Cardamine* and *Rorippa* species, indicating that this boundary site carries strong phylogenetic signal within the genus *Cardamine* and can serve as a potential molecular marker for species identification within the genus (Tutuş et al. 2025).

A total of 70 SSR loci were identified in the chloroplast genome of *C. microzyga*, among which mononucleotide repeats were absolutely dominant (81.4%), and all consisted entirely of A or T bases. This A/T bias is commonly observed in plant chloroplast genomes and may be related to the replication and repair mechanisms of chloroplast DNA (Kadam et al. 2025; Liu et al. 2025). Similar to the results of this study, SSR loci predominantly composed of A/T were also detected in the chloroplast genomes of *C. fallax* and *C. amariformis* (Raman and Park 2021; Raman et al. 2021). Nine compound SSRs were identified in this study, among which the motif of ID 59 was (A)10(AT)6*aa(T)11, exhibiting a unique structural feature. Compound SSRs generally possess higher mutation potential and may serve as high‐resolution molecular markers. This is because their longer, more complex structure increases the likelihood of replication slippage and secondary structure (Kadam et al. 2025). These newly identified SSR loci provide a wealth of candidate marker resources for future studies on genetic diversity assessment, population genetic structure analysis, and species identification within the genus *Cardamine*.

The chloroplast genome of *C. microzyga* contains 30 codons with an RSCU value greater than 1, the vast majority of which end with A or U, exhibiting a typical A/T bias. This phenomenon is consistent with the overall low GC content of the chloroplast genome and suggests that mutational pressure plays a dominant role in shaping the codon usage pattern (Yang et al. 2024). Furthermore, TAA is the most frequently used termination codon, which is consistent with findings from most angiosperm chloroplast genomes (Li et al. 2016). Leucine (Leu), arginine (Arg), and serine (Ser) are the most abundant amino acids, a feature commonly observed in other Brassicaceae species as well (Raman and Park 2022), which may be related to the high abundance of these amino acids in photosynthesis‐related proteins.

In this study, a total of nine hypervariable genes with Pi values exceeding 0.0156 were identified through the analysis of 106 protein‐coding genes, among which *trnS‐GCU* (Pi = 0.03,736), *rrn4.5S* (0.03,280), and *ycf1* (0.03,224) exhibited the highest levels of variation. The *ycf1* gene has been widely demonstrated to be one of the most significantly variable genes in chloroplast genomes and can serve as a candidate region for DNA barcoding (Dong et al. 2012; Amar 2020). *matK* (Pi = 0.02,212), as one of the core candidate fragments for plant DNA barcoding, also showed a relatively high level of variation in this study. In addition, 15 genes (including several tRNA genes and *rrn5S*) were completely conserved (Pi = 0) among the compared species, suggesting that these genes may be under strong functional constraints. It should be noted that these proposed barcode candidates are based on a preliminary screening of sequence divergence and require further validation using population‐level sampling and experimental assessment. The identification of the above hypervariable and conserved regions provides important references for future molecular systematics studies and DNA barcode development in the genus *Cardamine* and the Brassicaceae family.

Based on the complete chloroplast genomes of 45 Brassicaceae species, a maximum likelihood (ML) phylogenetic tree was constructed, which confirmed the monophyly of the genus *Cardamine* with high support (bootstrap = 100), consistent with previous findings (Ren et al. 2018). Within the genus *Cardamine*, *C. microzyga* was placed in Clade B, forming a highly supported subclade with *C. lyrata* , *C. fallax* , and *C. amariformis*, indicating that *C. microzyga* shares a recent common ancestor with these three species. *Cardamine* forms a sister group with *Rorippa*, and together with *Barbarea* they constitute a large clade. This topological structure is consistent with the results of combined analyses based on nuclear and chloroplast genes (Ajmal Ali et al. 2010), further supporting the close evolutionary relationships among these three genera within the Brassicaceae. The phylogenetic tree constructed in this study provides new chloroplast genomic evidence for the evolutionary relationships of the genus *Cardamine* and the Brassicaceae family.

## Author Contributions

**Yongming Fan:** conceptualization (equal), investigation (equal), methodology (equal), project administration (equal), validation (equal), writing – original draft (equal), writing – review and editing (equal). **Yan Wu:** data curation (equal), methodology (equal), validation (equal), writing – original draft (equal), writing – review and editing (equal). **Heng Zeng:** data curation (equal), writing – original draft (equal), writing – review and editing (equal). **Can Zhao:** data curation (equal), writing – original draft (equal), writing – review and editing (equal). **Chu Li:** data curation (equal), visualization (equal), writing – original draft (equal), writing – review and editing (equal). **Le Chen:** data curation (equal), formal analysis (equal), software (equal), validation (equal), visualization (equal), writing – original draft (equal), writing – review and editing (equal). **Jian Hu:** conceptualization (equal), data curation (equal), formal analysis (equal), writing – original draft (equal), writing – review and editing (equal). **Lingjun Li:** data curation (equal), funding acquisition (equal), project administration (equal), writing – original draft (equal), writing – review and editing (equal).

## Funding

This research was supported by the Natural Science Foundation of Henan (Grant number 252300423627), and the High‐level Talent Scientific Research Startup Project of North China University of Water Resources and Electric Power (Grant number 202110007).

## Conflicts of Interest

The authors declare no conflicts of interest.

## Supporting information


**Table S1:** The lengths of introns and exons for the splitting genes.

**Table S2:** Simple sequence repeats (SSR) in the *C. microzyga* cp genome.

**Table S3:** Codon usage and codon‐anticodon recognition patterns of *C. microzyga*.

## Data Availability

The original data of chloroplast genome *C. microzyga* has been deposited into CNSA with accession number CNX1361155.
